# Evaluation of Functional Decline in Alzheimer’s Dementia Using 3D Deep Learning and Group ICA for rs-fMRI Measurements

**DOI:** 10.3389/fnagi.2019.00008

**Published:** 2019-02-11

**Authors:** Muhammad Naveed Iqbal Qureshi, Seungjun Ryu, Joonyoung Song, Kun Ho Lee, Boreom Lee

**Affiliations:** ^1^Department of Biomedical Science and Engineering (BMSE), Institute of Integrated Technology (IIT), Gwangju Institute of Science and Technology (GIST), Gwangju, South Korea; ^2^Translational Neuroimaging Laboratory, The McGill University Research Centre for Studies in Aging, McGill University, Montreal, QC, Canada; ^3^Alzheimer’s Disease Research Unit, Douglas Mental Health University Institute, McGill University, Montreal, QC, Canada; ^4^Department of Psychiatry, McGill University, Montreal, QC, Canada; ^5^National Research Center for Dementia, Chosun University, Gwangju, South Korea; ^6^Department of Biomedical Science, Chosun University, Gwangju, South Korea

**Keywords:** dementia, progression assessment, imaging biomarkers, independent component analysis, neuroimaging, convolutional neural network

## Abstract

**Purpose**: To perform automatic assessment of dementia severity using a deep learning framework applied to resting-state functional magnetic resonance imaging (rs-fMRI) data.

**Method**: We divided 133 Alzheimer’s disease (AD) patients with clinical dementia rating (CDR) scores from 0.5 to 3 into two groups based on dementia severity; the groups with very mild/mild (CDR: 0.5–1) and moderate to severe (CDR: 2–3) dementia consisted of 77 and 56 subjects, respectively. We used rs-fMRI to extract functional connectivity features, calculated using independent component analysis (ICA), and performed automated severity classification with three-dimensional convolutional neural networks (3D-CNNs) based on deep learning.

**Results**: The mean balanced classification accuracy was 0.923 ± 0.042 (*p* < 0.001) with a specificity of 0.946 ± 0.019 and sensitivity of 0.896 ± 0.077. The rs-fMRI data indicated that the medial frontal, sensorimotor, executive control, dorsal attention, and visual related networks mainly correlated with dementia severity.

**Conclusions**: Our CDR-based novel classification using rs-fMRI is an acceptable objective severity indicator. In the absence of trained neuropsychologists, dementia severity can be objectively and accurately classified using a 3D-deep learning framework with rs-fMRI independent components.

## Introduction

Alzheimer’s disease (AD) is the most common form of dementia among dementia patients, with a 40%–60% prevalence (Ferri et al., 2005). It is a devastating illness and results in major cognitive and behavioral impairments. The established underlying mechanism is neurodegeneration, which is attributed to the accumulation of Aβ, hyperphosphorylation of tau proteins, and neuroinflammation (Leuner et al., 2007; Frautschy and Cole, 2010; Shadfar et al., 2015). Although molecular chemistry research on the mechanism of dementia has been conducted, numerous reports suggest structural and functional changes in the brain identified using neuroimaging (He et al., 2007; Solé-Padullés et al., 2009; Adlard et al., 2014). The assessment and treatment for patients with AD are multi-modal and are based on the stage of the illness. At each stage, the physician should alert and help the patients and their families to anticipate future symptoms and the related care that may be required. Although dementia symptoms can be controlled, slowing disease progression down is not a direct treatment for the pathophysiological mechanism of AD (Cummings and Fox, 2017). Given that most drugs currently used for treatment of AD patients act by enhancing cholinergic transmission and thus require viable synapses (DeKosky and Scheff, 1990; Terry and Buccafusco, 2003; Sarter and Parikh, 2005; Cacabelos, 2007), evaluation of the stage of dementia by experts is important for appropriate symptom control; additionally, the evaluation of viable synapse is important for determining the progression of the disease (DeKosky and Scheff, 1990; Scheff et al., 1990).

However, despite numerous neuroimaging studies, staging of dementia is generally based on past history and mental status examination by trained neuro-psychiatrists under the guidelines of the clinical dementia rating scale (CDR; Hughes et al., 1982). CDR helps the clinicians to rate the severity of AD and related disorders on a scale from 0 (normal) to 3 (severe stage) based on clinical interviews with a caregiver and the person with dementia. The areas that are coded are memory, orientation, judgment, problem-solving, community affairs, home, and hobbies. Despite the use of CDR, which is consensual among neuro-psychiatrists, and is based on extensive research and statistics to ensure the validity of the dementia severity rating, the diagnostic process mainly depends on the assessment of clinical symptoms. Furthermore, the diagnostic criteria of AD involves a substantial observation period and a reliable informant. In addition, it is too burdensome for a general doctor to use CDR (Perneczky et al., 2006). Also, CDR may have limitations in detecting early dementia (Rockwood et al., 2000; Schafer et al., 2004). Therefore, an additional tool for rating dementia severity is definitely required, and neuroimaging techniques may serve to complement the CDR scale.

Recently, resting-state functional connectivity is regarded as an important biomarker for AD. Several studies have reported that AD patients show decreased resting-state functional connectivity in the default mode network (DMN; Greicius et al., 2004; Hafkemeijer et al., 2012; Koch et al., 2012; Franciotti et al., 2013; Krajcovicova et al., 2014; Joo et al., 2016). Although atrophy was not observed, mild cognitive impairment (MCI) was associated with decreased functional connectivity of the medial temporal lobe or DMN region (Jin et al., 2012). Several resting-state functional magnetic resonance imaging (rs-fMRI) studies have addressed the issues of early detection, classification, and prediction in AD, MCI, normal patients, and subtypes of dementia. Previous reports have provided optimistic results for the classification of AD, MCI, and healthy normal aging individuals. Various approaches, such as independent component analysis (ICA; Fox et al., 2006; Dosenbach et al., 2007; Sylvester et al., 2009; Zhou et al., 2010), region of interest (Wang et al., 2006; Chen et al., 2011; Challis et al., 2015), graph theory (Supekar et al., 2008; Khazaee et al., 2015), multivoxel pattern analysis using machine learning (Mahmoudi et al., 2012), and multimodal (Dai et al., 2012; Dyrba et al., 2015) approaches have shown high performance (72%–94% accuracy). However, most prior studies have used datasets only from a single site/source, except for a study in which the AD neuroimaging initiative (ADNI) dataset was compared to their in-house dataset for validation of MCI/Normal classification algorithm (Suk et al., 2016). Therefore, the classification format of most previous studies strictly followed the form of the database. The ADNI dataset is aimed at early detection of AD, and related studies focus on classifying the normal patients, MCI, and early AD. Therefore, ADNI did not contain adequate numbers of severe-stage patients diagnosed with CDR 2 or 3 score (late AD).

ICA is an effective method for functional connectivity analysis of brain imaging data (Lu and Rajapakse, 2006; Rajapakse and Zhou, 2007; Brier et al., 2012). Previously, numerous studies have reported greater functional connectivity in the salience (SAL) of patients with mild dementia (primarily CDR 1) than in normal individuals (Fox et al., 2006; Dosenbach et al., 2007; Sylvester et al., 2009; Zhou et al., 2010). In contrast, functional connectivity increments of the SAL were seen at levels between CDR 0 and CDR 0.5, which implicates a reduced correlation at CDR 1. This difference depends on the method used to acquire the independent components (Brier et al., 2012). In the past, the ICA components were reviewed by trained clinicians for the selection of meaningful components (Oh et al., 2017). Currently, ICA components can be automatically selected using highly advanced algorithms (Beckmann et al., 2009; Filippini et al., 2009). On applying these algorithms, we can consistently and automatically select the ICA components in classification studies.

Deep learning has gained enormous attention (Gal and Ghahramani, 2016; Amiri et al., 2018) in the last few years. The recent advances in machine learning in terms of image understanding have led to great advances with respect to identifying, classifying, and quantifying patterns of medical images, especially using deep learning. In particular, the utilization of hierarchical functional representations learned solely with data, instead of manually created features that are designed based on domain-specific knowledge is at the core of the progress (Raju et al., 2017; Shen et al., 2017; Amiri et al., 2018). Previous studies have reported that the classification of dementia, MCI, and normal individuals can be performed automatically using deep learning and multimodal data including neuroimaging data or biological measures from cerebrospinal fluid (CSF; Suk and Shen, 2013; Liu et al., 2015; Suk et al., 2015). Automated diagnostics using multimodal neuroimaging data have the advantage of utilizing all information, and demonstrate the potential to improve diagnostic accuracy. However, the process is highly complex and requires additional computational resources. Therefore, it would be preferable to obtain acceptable accuracy with only unimodal data.

Three-dimensional convolutional neural network (3D-CNN) in deep learning is a supervised learning framework and is enabled to distinguish training data similar to the visual processing of the human eye (Ji et al., 2013). While these networks have been used specifically for visual recognition in the 2D domain over the last few years by researchers in visual computing and artificial intelligence research, it is unlikely that 3D-CNN was used for volumetric neuroimaging data classification and prediction. The novelty of this study is that 3D ICA data were used as input for the 3D-CNN model. Considering that previous studies have shown that group ICA features have the potential to discriminate dementia severity, we classified the severity of dementia using 3D deep learning with group ICA input.

Despite its clinical importance, the severity estimation of AD using image data was not conducted by any researcher at all, except for one report that characterizes five resting state networks of CDR 0.5 and 1 (Brier et al., 2012). Therefore, a major novel feature of our research is the automatic classification of AD into two groups of disease severity (very mild and mild vs. moderate and severe).

To propose an alternative method to complement the CDR scale in the evaluation of AD, we hypothesized that the functional connectivity changes according to the stage of AD will be observed in the rs-fMRI, and the severity of AD could be classified using 3D-CNN.

## Materials and Methods

### Dataset

This dataset was a part of a large cohort enrolled at National Dementia Research Center, Chosun University, Gwangju, South Korea. Each subject provided written informed consent before the data collection. The data acqusistion was approved by the institutional review board of the Chosun University Hospital, Gwangju, South Korea (IRB number 2013-12-018).

The demographics of the participants are shown in Table 1. CDR is a categorical variable. To better estimate the decline of resting-state functional connectivity with increasing AD severity, we allocated the labeled data into two groups. Group 1 includes very mild to mild (CDR 0.5 and 1.0) and group 2 includes moderate to severe (CDR 2.0–3.0) patients.

**Table 1:** Subject demographics.

	Very mild to mild AD	Moderate to severe AD	
	(*n* = 77; 30 F/47 M)	(*n* = 49; 32 F/17 M)	*p*-value
Age (years)	73.57 ± 6.49	73.61 ± 4.76	0.160
Education (score)	10.09 ± 4.95	6.79 ± 4.54	0.227
MMSE (score)	23.84 ± 3.90	15.49 ± 4.87	0.09
CDR (score)	0.71 ± 0.25	2.08 ± 0.28	0.001*

*The p-value was computed by applying the t-test to the clinical dementia rating (CDR) scores*.

### Resting-State fMRI Data Acquisition

All the participants were scanned with a Siemens Skyra 3.0-Tesla scanner. A 2D EPI MR acquisition type was used with the following parameters: TR/TE = 3,000/30 ms, flip angle = 90°, FOV = 240 × 240 mm, voxel size = 3.75 × 3.75 × 3.75, spacing between slices = 4.8 mm, number of echoes = 1, imaging frequency = 123.206 Hz, slice acquisition order = ascending (bottom-up), direction = ‘Transverse > Coronal (2.6) > Saggital (1.7)’, pixel bandwidth = 3440, inplane phase encoding direction = ‘ROW’, number of phase encoding steps = 63, echo train length = 31° sampling = 100° phase field of view = 100, variable flip angle flag = ‘N’, and SAR = 0.0778.

### Preprocessing of the Resting-State fMRI Data

**Figure 1:**
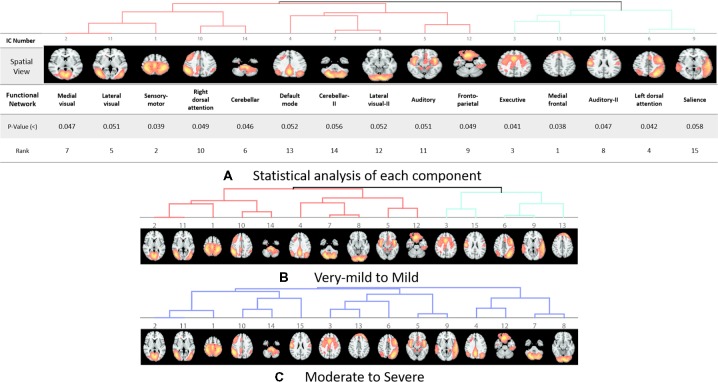
Dendrograms of the selected useful independent component-based functional networks using automated clustering. **(A)** Statistical analysis of each component. **(B)** Dendrogram of very-mild and mild groups, two major divisions are shown in red and green color. **(C)** Dendrogram of moderate and severe groups, no significant division was found among the functional networks according to the clinical dementia rating (CDR) level.

The rs-fMRI data was pre-processed with FMRIB Software Library (FSL^1^) version 6.0. Standard preprocessing routines were applied with motion correction, slice timing correction, spatial smoothing with 6 mm full width half maximum Gaussian kernel, temporal filtering, and thereafter each subject’s functional data were co-registered to its corresponding structural image. Subsequently, for acquiring the group ICA based connectivity measures, FSL Multivariate Exploratory Linear Optimized Decomposition into Independent Components (MELODIC) version 3.14 was utilized to perform a single-session ICA. The number of independent components was set as 30 (Qureshi et al., 2017). We used variance normalization and thresholded the independent component maps with an alternative hypothesis test that was based on the fitting of a Gaussian/gamma mixture model to the distributions of the voxel intensities within the spatial maps and controlling the local false-discovery rate at *p* < 0.5. The set of spatial maps from the group-average analysis was used to generate subject-specific versions of the spatial maps, and associated time-series, using dual regression (Beckmann et al., 2005, 2009). First, for each subject, the group-average set of spatial maps is regressed (as spatial regressors in a multiple regression) into the subject’s 4D space-time dataset (Oh et al., 2017; Qureshi et al., 2017). This results in a set of subject-specific time-series, one per group-level spatial map. Next, those time series are regressed (as temporal regressors, again in a multiple regression) into the same 4D dataset, resulting in a set of subject-specific 3D spatial maps, one per group-level. We then tested for group differences, using FSL’s randomized permutation-testing tool (Smith et al., 2004). Among the 30 independent components, 15 were classified as noise and/or artifacts using the automated clustering tool of FSLNets^2^. Besides the automated selection, these components were also validated by visual inspection by an experienced clinical neurologist, similar to the procedure used in our previous studies (Qureshi et al., 2017). Figure 1A depicts the selected 15 components. It represents the well-known resting-state functional networks including the DMN, sensorimotor network, medial and lateral visual network, left and right dorsal attention network, central executive network, cerebellar network, salience network, limbic network, auditory network, and frontal networks.

### Features

We used the 3D volumetric images of these selected functional networks for the classification between the CDR low and CDR high groups. These 3D images were acquired by performing dual regression (Beckmann et al., 2009) on the group ICA result.

### Deep Learning and 3D-CNN Framework

**Figure 2:**
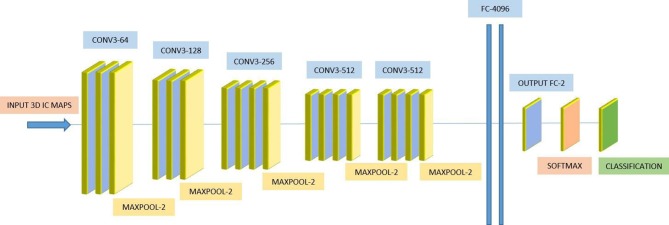
VGG-Net based three-dimensional convolutional neural network (3D-CNN) architecture.

We used a 3D-CNN based deep learning classification framework in this study. This framework was implemented on the TensorFlow library version 1.5 with Nvidia Geforce GTX 1080Ti graphical processing unit (GPU) support. For the training model, we used the Adam optimizer with a learning rate of 0.001, epsilon value was set at 0.1, and minimal cost was used. Since the size of the dataset was relatively small for deep learning, to avoid model overfit, we used ten-fold cross-validation in this study to report the mean accuracy of the model. A modified version of VGG-Net classification framework was used in this study. Specifically, we added batch normalization layers in the convolution layer. A dropout rate of 0.7 was used in the fully connected layers. The batch size was set at 12 and 50 epochs were used. The parameters including learning rate, epsilon value, dropout rate, batch size, and epoch size were optimized using the following ranges. For epsilon, we tunned it in the range of [0.1 : 0.05 : 1], for learning rate, we tunned it in the logarithmic range of [1, 0.1, 0.01, 0.001, 0.0001, and 0.00001], for the dropout rate, we tunned it in the range [0.1 : 0.05 : 1], for the batch size, we optimized it by the maximum available GPU memory, and the number of epochs were tunned in the range of [10 : 1 : 200]. To the best of our knowledge, CNN is the only deep learning framework that learn from 3D input, therefore no other deep learning architectures were tested during this study. Figure 2 depicts the complete architecture of our 3D-CNN deep classification framework. Details of the model are given in Table 2.

**Table 2:** Details of the three-dimensional convolutional neural network (3D-CNN) architecture.

Layer	Feature Map	Stride	Kernel	Activation structure
Convolution	64	1 × 1 × 1	3 × 3 × 3	Conv
Convolution	64	1 × 1 × 1	3 × 3 × 3	Batchnorm+ReLU+Conv
Maxpool		2 × 2 × 2	2 × 2 × 2	
Convolution	128	1 × 1 × 1	3 × 3 × 3	Batchnorm+ReLU+Conv
Convolution	128	1 × 1 × 1	3 × 3 × 3	Batchnorm+ReLU+Conv
Maxpool		1 × 1 × 1	2 × 2 × 2	
Convolution	256	1 × 1 × 1	3 × 3 × 3	Batchnorm+ReLU+Conv
Convolution	256	1 × 1 × 1	3 × 3 × 3	Batchnorm+ReLU+Conv
Convolution	256	1 × 1 × 1	3 × 3 × 3	Batchnorm+ReLU+Conv
Maxpool		2 × 2 × 2	2 × 2 × 2	
Convolution	512	1 × 1 × 1	3 × 3 × 3	Batchnorm+ReLU+Conv
Convolution	512	1 × 1 × 1	3 × 3 × 3	Batchnorm+ReLU+Conv
Convolution	512	1 × 1 × 1	3 × 3 × 3	Batchnorm+ReLU+Conv
Maxpool		2 × 2 × 2	2 × 2 × 2	
Convolution	512	1 × 1 × 1	3 × 3 × 3	Batchnorm+ReLU+Conv
Convolution	512	1 × 1 × 1	3 × 3 × 3	Batchnorm+ReLU+Conv
Convolution	512	1 × 1 × 1	3 × 3 × 3	Batchnorm+ReLU+Conv
Maxpool		1 × 1 × 1	2 × 2 × 2	
Fully Connected	4096	Dropout rate 0.7		ReLU
Fully Connected	4096	Dropout rate 0.7		ReLU
**Output**				
Fully Connected	2			
Softmax				
Classification Layer	Argmax			

### Significance Testing

For assessing the statistical significance of the results, we performed the permutation test on the classification accuracies and permuted the labels of test data of each of the 10 folds 1,000 times to get the probability of successful classification with a higher score than the actual test labels.

## Results

Our results suggest that CDR level can be used as a good discriminatory predictor of the dementia stages. We achieved a mean balanced test accuracy of 92.30% in a ten-fold cross validation experiment using the 3D-CNN algorithm.

### Classification

We achieved an optimistic 10-fold cross-validated classification accuracy. Since the dataset was not balanced, we also computed the balanced accuracy to remove any bias present in the result due to unbalanced data. Table 3 shows all the performance evaluation measures in the data including the test accuracy, train accuracy, specificity, sensitivity, and balanced accuracy.

**Table 3:** Classification accuracy using 10-fold cross-validation.

Fold	Train Acc (%)	Test Acc (%)	*p*-value	AUC	Specificity (%)	Sensitivity (%)	BAC
1	99.44	95.83	<0.001	0.9936	0.9421	0.9871	0.9647
2	99.72	95.31	<0.001	0.9832	0.9561	0.9294	0.9428
3	99.94	91.66	<0.001	0.9838	0.9162	0.8864	0.9161
4	99.94	88.02	<0.001	0.9649	0.9320	0.8021	0.8671
5	99.94	91.14	<0.001	0.9767	0.9532	0.8587	0.9059
6	99.94	95.83	<0.001	0.9934	0.9734	0.9419	0.9577
7	99.04	94.79	<0.001	0.9809	0.9483	0.9398	0.9441
8	99.88	83.85	<0.001	0.9765	0.9456	0.7383	0.8419
9	99.61	92.18	<0.001	0.9740	0.9231	0.9146	0.9189
10	99.72	96.88	<0.001	0.9936	0.9739	0.9642	0.9690
Mean ± SD	99.72 ± 0.29	92.55 ± 4.11		0.982 ± 0.009	0.946 ± 0.019	0.896 ± 0.077	0.923 ± 0.042

### Statistical Significance

Statistically, this result has very high significance with *p* < 0.001 for all the 10-folds of the classification experiment. The significance measure through permutation testing were computhed as the *p*-values as mentioned in Table 3 for each fold of the cross-validation.

### Clinical Significance

**Figure 3:**
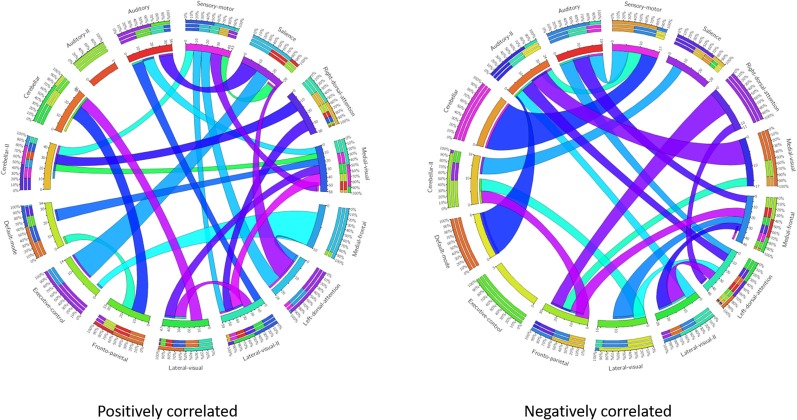
Connectogram of all the selected networks. Left connectogram shows the positively correlated connections among the functional networks. Right connectogram shows the negatively correlated connections.

These results suggest that CDR-based novel classification of rs-fMRI can be accepted as an objective severity index. Table 4 shows the ranking of each functional network as the features of a deep learning framework based on the unpaired *t*-test. The uncorrected *p*-value revealed the component’s significance. Figure 3 shows the connectogram of the selected networks.

**Table 4:** Statistical analysis of each component.

Component name	Component number	uncorrected *p*-value (<)	Rank
Sensory-motor network	1	0.038933	2
Medial visual-related network	2	0.046845	7
Executive control network	3	0.040517	3
Default mode network	4	0.052597	13
Auditory related network	5	0.051502	11
Left dorsal attention network	6	0.042201	4
Cerebellar network	7	0.056729	14
Lateral visual-related network	8	0.052143	12
Salience network	9	0.058392	15
Right dorsal attention network	10	0.049393	10
Lateral visual-related network-II	11	0.043619	5
Fronto-parietal network	12	0.049307	9
Medial frontal network	13	0.038227	1
Cerebellar network-II	14	0.045902	6
Auditory related network-II	15	0.047154	8

## Discussion

To the best of our knowledge, this is a pioneering study to classify the severity of dementia using rs-fMRI and 3D-CNN deep learning architecture rather than a 1D time-series information. Because the assessment of symptoms of patients with AD is important for appropriate treatment, the automatic classification of AD of the two groups of disease severity has important contributions for clinical practice.

There are previous studies on automated diagnosis using deep learning and multimodal neuroimaging data involving the CSF and laboratory assessments. Among these, there are numerous studies that classified dementia, MCI, and healthy individuals (Suk and Shen, 2013; Liu et al., 2015; Suk et al., 2015). It may be helpful to analyze structural MRI changes in distinguishing between normal patients, MCI, and AD. However, since structural changes are more likely to have progressed beyond a certain level, structural MRI may act as a confounding factor when considering individual differences. CSF studies may be helpful in assessing severity. To acquire CSF samples, we perform an invasive procedure, which is a lumbar puncture. However, considering the enviornment of out patient departments in Korean hospitals, it is diffciult to perform invasive procedures. Overall, if cost-effectiveness was taken into account, it would be best that the severity was determined using only noninvasive rs-fMRI. If only rs-fMRI was used, the imaging time could be less than a few minutes and may prove effective in clinical management.

Only one study reported the characteristics of five resting state networks of CDR score from 0.5 to 1 (Brier et al., 2012). This report provided clues to the discriminatory potential of group ICA features that could contribute to the classification of dementia severity. However, no study has been conducted on patients with CDR scores of 2 or 3 with ICA as features, which were classified automatically from noise using FSLNet and deep learning structure. Therefore, the major contribution of our research is the automatic classification of AD into two groups of disease severity (very mild and mild vs. moderate and severe).

Our results showed a mean test accuracy of 92.30% in a 10-fold cross validation experiment using the 3D-CNN algorithm. We believe that a deep neural network constitutes the optimal classification weight through iterative learning, but the extent of contribution of the ICA component of deep learning architecture to the algorithm is not known. To reveal the black box of 3D-CNN, we also compared each component between very mild/mild vs. moderate/ severe patients. Previous studies have reported that the DMN is the most significant different functional network between normal patients and MCI and dementia (Wang et al., 2006; Jin et al., 2012; Koch et al., 2012), and the salience network had differences between CDR 0.5 and 1 (Fox et al., 2006; Dosenbach et al., 2007; Sylvester et al., 2009; Zhou et al., 2010). Interestingly, our result showed that the medial frontal, sensory-motor, executive control, left dorsal attention, lateral visual-related, cerebellar, medial visual-related, auditory-related, frontoparietal, and right dorsal attention networks have high ranks and statistical differences. After the onset of dementia, functional connectivity seems to be observed in an altered way. We assumed that those networks have more influence on our classifier. Although DMN and salience network do not have enough statistical significance, the combination of the information from various components and their relationship including functional connectivity may contribute to the classification algorithm. Figures 1, 2 show the relationships among the components. Red color represents positive correlations and blue color represents negative correlation among the components. These associations represent the activity of each component, and there were no significant differences between the two groups, which is also shown in Table 4. Even in case of subtle differences, with deep learning these can be utilized to extract features to render the weights more suitable.

Research on drug development for AD has not been able to improve drug-based treatments, in spite of the recently advanced understanding of the molecular-cellular biology of the disease (De Strooper, 2014; Gauthier et al., 2016). Although, there may be numerous reasons for the failure of new drug development, as the stage of dementia differs from patient to patient, it is difficult to evaluate the response to symptoms alone. In addition, dementia could be a confounding factor due to the differences in the characteristics of individuals including genomic, proteomic, and metabolomic cascades. A previous study reported that current trials have focused on clinical efficacy and not on the rigorous testing of the putative mechanisms of disease (Becker et al., 2014). Considering that the central cholinergic deficit in AD is the consequence of neurodegeneration, the imaging method of measuring viable synapses is appropriate for evaluating drug responses. Because fMRI measures the function of the brain through the blood oxygen level dependent technique, it may help to compensate for the weaknesses of drug efficacy assessment through symptoms. Our classification algorithm based unimodal rs-fMRI extracts features from the degeneration of the functional connectivity in dementia. During the evaluation of drug response or behavioral therapy according to the stage and symptoms of AD, it would be helpful to investigate the recovery of functional connectivity objectively.

The novelty of our study is that we analyzed the severity of dementia, although our study also has limitations. We used our dataset to create a 3D-CNN classifier, but we could not perform the verification procedure with other datasets. Because of the ADNI dataset, which has been widely used in previous dementia studies, we could focus on early stage dementia detection; and the numbers of late-stage dementia patients were not adequate for comparison. It is necessary to apply our algorithm to other datasets with adequate numbers of patients with late-stage dementia.

Another limitation is due to the characteristics of deep learning. A total of 15 ICAs were selected as input for deep learning, but it is difficult to determine the precise effect on the neural network. To overcome this limitation, we statistically analyzed the differences of ICA between the two groups.

Another limition of the present study is in terms of the limited number of subjects, however, it is inappropriate to apply standard data augmentation approaches on the neuroimaging data to increase the number of training samples. We believe that the introduction of any type of synthesized data in training phase can significantly bias the learning process. In addition, the signal to noise ratio in fMRI data is relatively small therefore it is very difficult to apply deep learning to the raw data. A major advantage of using ICA is the removal of artifacts because they very much look like the BOLD signal in raw data.

One of the most important aspects of this research is the use of neuroimaging to predict the progression of diseases that humans can not predict, especially for the subjects with MCI who progress to dementia as compared to those who do not progress to dementia in the future. However, we cannot represent it in our present study. The classification task in this research has a limitation because the data labels used in this study are based on contemporary clinical evaluations. In addition, classifying current disease status is important but clinically, predicting the progression from MCI to dementia and classifying severity in dementia is more important for proper and appropriate treatment, and also prediction from MCI to dementia and current severity classification can have a decisive impact on prognosis. Taking all of these measures into account, our analysis can be considered as a clinically relevant study involving future outcomes. In the future, we will also perform an advanced study to predict the progression from MCI to dementia using biomarker-based serial labeled data and domain transfer learning methods.

In conclusion, our study suggests that our novel classifier using rs-fMRI is acceptable as an objective severity indicator complementing the CDR scale in the evaluation of AD. In the absence of trained neurologists, we can classify the dementia severity objectively and accurately using 3D-deep learning. Our application and classification algorithm would be an aid for observing the regeneration of functional connectivity due to drug treatment according to the stage and symptoms of AD in the future.

## Data Availability

The datasets for this study will not be made publicly available because this study cohort is not open for public use.

## Author Contributions

MQ and SR have equally contributed in this work. MQ developed the whole idea of this work. SR made the clinical representation of the results. JS helped in the design of deep learning framework. KL and BL supervised the research.

## Conflict of Interest Statement

The authors declare that the research was conducted in the absence of any commercial or financial relationships that could be construed as a potential conflict of interest.

## Footnotes

^1^www.fmrib.ox.ac.uk/fsl

^2^https://fsl.fmrib.ox.ac.uk/fsl/fslwiki/FSLNets

## Abbreviations

3D-CNN: three-dimensional convolutional neural networks
CDR: clinical dementia rating
CSF: cerebrospinal fluid
ICA: independent component analysis
rs-fMRI: resting-state functional magnetic resonance imaging
MCI: mild cognitive impairment.

## References

[B1] Adlard P. A. Tran B. A. Finkelstein D. I. Desmond P. M. Johnston L. A. Bush A. I.. (2014). A review of β-amyloid neuroimaging in Alzheimer’s disease. Front. Neurosci. 8:327. 10.3389/fnins.2014.00327 25400539 PMC4215612

[B2] Amiri S. Mahjoub M. A. Rekik I. (2018). “Bayesian network and structured random forest cooperative deep learning for automatic multi-label brain tumor segmentation,” in Proceedings of the 10th International Conference on Agents and Artificial Intelligence (ICAART 2018)—Volume 2, 183–190.

[B3] Becker R. E. Greig N. H. Giacobini E. Schneider L. S. Ferrucci L. (2014). A new roadmap for drug development for Alzheimer’s disease. Nat. Rev. Drug Discov. 13:156. 10.1038/nrd3842-c2 24362362 PMC4668586

[B4] Beckmann C. F. DeLuca M. Devlin J. T. Smith S. M. (2005). Investigations into resting-state connectivity using independent component analysis. Philos. Trans. R. Soc. Lond. B Biol. Sci. 360, 1001–1013. 10.1098/rstb.2005.1634 16087444 PMC1854918

[B5] Beckmann C. F. Mackay C. E. Filippini N. Smith S. M. (2009). Group comparison of resting-state FMRI data using multi-subject ICA and dual regression. Neuroimage 47:S148 10.1016/s1053-8119(09)71511-3

[B6] Brier M. R. Thomas J. B. Snyder A. Z. Benzinger T. L. Zhang D. Raichle M. E.. (2012). Loss of intranetwork and internetwork resting state functional connections with Alzheimer’s disease progression. J. Neurosci. 32, 8890–8899. 10.1523/JNEUROSCI.5698-11.2012 22745490 PMC3458508

[B7] Cacabelos R. (2007). Donepezil in Alzheimer’s disease: from conventional trials to pharmacogenetics. Neuropsychiatr. Dis. Treat. 3:303. 19300564 PMC2654795

[B8] Challis E. Hurley P. Serra L. Bozzali M. Oliver S. Cercignani M. (2015). Gaussian process classification of Alzheimer’s disease and mild cognitive impairment from resting-state fMRI. Neuroimage 112, 232–243. 10.1016/j.neuroimage.2015.02.037 25731993

[B9] Chen G. Ward B. D. Xie C. Li W. Wu Z. Jones J. L.. (2011). Classification of Alzheimer disease, mild cognitive impairment and normal cognitive status with large-scale network analysis based on resting-state functional MR imaging. Radiology 259, 213–221. 10.1148/radiol.10100734 21248238 PMC3064820

[B10] Cummings J. Fox N. (2017). Defining disease modifying therapy for Alzheimer’s disease. J. Prev. Alzheimers Dis. 4, 109–115. 10.14283/jpad.2017.12 29071250 PMC5653310

[B11] Dai Z. Yan C. Wang Z. Wang J. Xia M. Li K.. (2012). Discriminative analysis of early Alzheimer’s disease using multi-modal imaging and multi-level characterization with multi-classifier (M3). Neuroimage 59, 2187–2195. 10.1016/j.neuroimage.2011.10.003 22008370

[B12] De Strooper B. (2014). Lessons from a failed γ-secretase Alzheimer trial. Cell 159, 721–726. 10.1016/j.cell.2014.10.016 25417150

[B13] DeKosky S. T. Scheff S. W. (1990). Synapse loss in frontal cortex biopsies in Alzheimer’s disease: correlation with cognitive severity. Ann. Neurol. 27, 457–464. 10.1002/ana.410270502 2360787

[B14] Dosenbach N. U. F. Fair D. A. Miezin F. M. Cohen A. L. Wenger K. K. Dosenbach R. A. T.. (2007). Distinct brain networks for adaptive and stable task control in humans. Proc. Natl. Acad. Sci. U S A 104, 11073–11078. 10.1073/pnas.0704320104 17576922 PMC1904171

[B15] Dyrba M. Grothe M. Kirste T. Teipel S. J. (2015). Multimodal analysis of functional and structural disconnection in Alzheimer’s disease using multiple kernel SVM. Hum. Brain Mapp. 36, 2118–2131. 10.1002/hbm.22759 25664619 PMC6869829

[B16] Ferri C. P. Prince M. Brayne C. Brodaty H. Fratiglioni L. Ganguli M.. (2005). Global prevalence of dementia: a Delphi consensus study. Lancet 366, 2112–2117. 10.1016/S0140-6736(05)67889-0 16360788 PMC2850264

[B17] Filippini N. MacIntosh B. J. Hough M. G. Goodwin G. M. Frisoni G. B. Smith S. M.. (2009). Distinct patterns of brain activity in young carriers of the APOE-ε4 allele. Proc. Natl. Acad. Sci. U S A 106, 7209–7214. 10.1073/pnas.0811879106 19357304 PMC2678478

[B18] Fox M. D. Corbetta M. Snyder A. Z. Vincent J. L. Raichle M. E. (2006). Spontaneous neuronal activity distinguishes human dorsal and ventral attention systems. Proc. Natl. Acad. Sci. U S A 103, 10046–10051. 10.1073/pnas.0606682103 16788060 PMC1480402

[B19] Franciotti R. Falasca N. W. Bonanni L. Anzellotti F. Maruotti V. Comani S.. (2013). Default network is not hypoactive in dementia with fluctuating cognition: an Alzheimer disease/dementia with Lewy bodies comparison. Neurobiol. Aging 34, 1148–1158. 10.1016/j.neurobiolaging.2012.09.015 23063646

[B20] Frautschy S. A. Cole G. M. (2010). Why pleiotropic interventions are needed for Alzheimer’s disease. Mol. Neurobiol. 41, 392–409. 10.1007/s12035-010-8137-1 20437209 PMC2876259

[B21] Gal Y. Ghahramani Z. (2016). “Dropout as a Bayesian approximation: representing model uncertainty in deep learning,” in Proceedings of The 33rd International Conference on Machine Learning (New York, NY: ACM), 1050–1059.

[B22] Gauthier S. Albert M. Fox N. Goedert M. Kivipelto M. Mestre-Ferrandiz J.. (2016). Why has therapy development for dementia failed in the last two decades? Alzheimers Dement. 12, 60–64. 10.1016/j.jalz.2015.12.003 26710325

[B23] Greicius M. D. Srivastava G. Reiss A. L. Menon V. (2004). Default-mode network activity distinguishes Alzheimer’s disease from healthy aging: evidence from functional MRI. Proc. Natl. Acad. Sci. U S A 101, 4637–4642. 10.1073/pnas.0308627101 15070770 PMC384799

[B24] Hafkemeijer A. van der Grond J. Rombouts S. A. R. B. (2012). Imaging the default mode network in aging and dementia. Biochim. Biophys. Acta 1822, 431–441. 10.1016/j.bbadis.2011.07.008 21807094

[B25] He Y. Wang L. Zang Y. Tian L. Zhang X. Li K.. (2007). Regional coherence changes in the early stages of Alzheimer’s disease: a combined structural and resting-state functional MRI study. Neuroimage 35, 488–500. 10.1016/j.neuroimage.2006.11.042 17254803

[B26] Hughes C. P. Berg L. Danziger W. L. Coben L. A. Martin R. L. (1982). A new clinical scale for the staging of dementia. Br. J. Psychiatry 140, 566–572. 10.1192/bjp.140.6.566 7104545

[B27] Ji S. Yang M. Yu K. (2013). 3D convolutional neural networks for human action recognition. IEEE Trans. Pattern Anal. Mach. Intell. 35, 221–2231. 10.1109/tpami.2012.59 22392705

[B28] Jin M. Pelak V. S. Cordes D. (2012). Aberrant default mode network in subjects with amnestic mild cognitive impairment using resting-state functional MRI. Magn. Reson. Imaging 30, 48–61. 10.1016/j.mri.2011.07.007 21982164 PMC3232317

[B29] Joo S. H. Lim H. K. Lee C. U. (2016). Three large-scale functional brain networks from resting-state functional MRI in subjects with different levels of cognitive impairment. Psychiatry Investig. 13, 1–7. 10.4306/pi.2016.13.1.1 26766941 PMC4701672

[B30] Khazaee A. Ebrahimzadeh A. Babajani-Feremi A. (2015). Identifying patients with Alzheimer’s disease using resting-state fMRI and graph theory. Clin. Neurophysiol. 126, 2132–2141. 10.1016/j.clinph.2015.02.060 25907414

[B31] Koch W. Teipel S. Mueller S. Benninghoff J. Wagner M. Bokde A. L. W.. (2012). Diagnostic power of default mode network resting state fMRI in the detection of Alzheimer’s disease. Neurobiol. Aging 33, 466–478. 10.1016/j.neurobiolaging.2010.04.013 20541837

[B32] Krajcovicova L. Marecek R. Mikl M. Rektorova I. (2014). Disruption of resting functional connectivity in Alzheimer’s patients and at-risk subjects. Curr. Neurol. Neurosci. Rep. 14:491. 10.1007/s11910-014-0491-3 25120223

[B33] Leuner K. Hauptmann S. Abdel-Kader R. Scherping I. Keil U. Strosznajder J. B.. (2007). Mitochondrial dysfunction: the first domino in brain aging and Alzheimer’s disease? Antioxid. Redox Signal. 9, 1659–1675. 10.1089/ars.2007.1763 17867931

[B34] Liu S. Liu S. Cai W. Che H. Pujol S. Kikinis R.. (2015). Multimodal neuroimaging feature learning for multiclass diagnosis of Alzheimer’s disease. IEEE Trans. Biomed. Eng. 62, 1132–1140. 10.1109/tbme.2014.2372011 25423647 PMC4394860

[B35] Lu W. Rajapakse J. C. (2006). ICA with reference. Neurocomputing 69, 2244–2257. 10.1016/j.neucom.2005.06.021

[B36] Mahmoudi A. Takerkart S. Regragui F. Boussaoud D. Brovelli A. (2012). Multivoxel pattern analysis for FMRI data: a review. Comput. Math. Methods Med. 2012:961257. 10.1155/2012/961257 23401720 PMC3529504

[B37] Oh J. Chun J.-W. Kim E. Park H.-J. Lee B. Kim J.-J. (2017). Aberrant neural networks for the recognition memory of socially relevant information in patients with schizophrenia. Brain Behav. 7:e00602. 10.1002/brb3.602 28127520 PMC5256185

[B38] Perneczky R. Wagenpfeil S. Komossa K. Grimmer T. Diehl J. Kurz A. (2006). Mapping scores onto stages: mini-mental state examination and clinical dementia rating. Am. J. Geriatr. Psychiatry 14, 139–144. 10.1097/01.jgp.0000192478.82189.a8 16473978

[B39] Qureshi M. N. I. Oh J. Cho D. Jo H. J. Lee B. (2017). Multimodal discrimination of schizophrenia using hybrid weighted feature concatenation of brain functional connectivity and anatomical features with an extreme learning machine. Front. Neuroinform. 11:59. 10.3389/fninf.2017.00059 28943848 PMC5596100

[B40] Rajapakse J. C. Zhou J. (2007). Learning effective brain connectivity with dynamic Bayesian networks. Neuroimage 37, 749–760. 10.1016/j.neuroimage.2007.06.003 17644415

[B41] Raju M. Pagidimarri V. Barreto R. Kadam A. Kasivajjala V. Aswath A. (2017). Development of a deep learning algorithm for automatic diagnosis of diabetic retinopathy. Stud. Health Technol. Inform. 245, 559–563. 29295157

[B42] Rockwood K. Strang D. MacKnight C. Downer R. Morris J. C. (2000). Interrater reliability of the Clinical Dementia Rating in a multicenter trial. J. Am. Geriatr. Soc. 48, 558–559. 10.1111/j.1532-5415.2000.tb05004.x 10811551

[B43] Sarter M. Parikh V. (2005). Choline transporters, cholinergic transmission and cognition. Nat. Rev. Neurosci. 6, 48–56. 10.1038/nrn1588 15611726

[B44] Schafer K. A. Tractenberg R. E. Sano M. Mackell J. A. Thomas R. G. Gamst A.. (2004). Reliability of monitoring the clinical dementia rating in multicenter clinical trials. Alzheimer Dis. Assoc. Disord. 18, 219–222. 15592134 PMC4367865

[B45] Scheff S. W. DeKosky S. T. Price D. A. (1990). Quantitative assessment of cortical synaptic density in Alzheimer’s disease. Neurobiol. Aging 11, 29–37. 10.1016/0197-4580(90)90059-9 2325814

[B46] Shadfar S. Hwang C. J. Lim M.-S. Choi D.-Y. Hong J. T. (2015). Involvement of inflammation in Alzheimer’s disease pathogenesis and therapeutic potential of anti-inflammatory agents. Arch. Pharm. Res. 38, 2106–2119. 10.1007/s12272-015-0648-x 26289122

[B47] Shen D. Wu G. Suk H.-I. (2017). Deep learning in medical image analysis. Annu. Rev. Biomed. Eng. 19, 221–248. 10.1146/annurev-bioeng-071516-044442 28301734 PMC5479722

[B48] Smith S. M. Jenkinson M. Woolrich M. W. Beckmann C. F. Behrens T. E. J. Johansen-Berg H.. (2004). Advances in functional and structural MR image analysis and implementation as FSL. Neuroimage 23, S208–S219. 10.1016/j.neuroimage.2004.07.051 15501092

[B49] Solé-Padullés C. Bartrés-Faz D. Junqué C. Vendrell P. Rami L. Clemente I. C.. (2009). Brain structure and function related to cognitive reserve variables in normal aging, mild cognitive impairment and Alzheimer’s disease. Neurobiol. Aging 30, 1114–1124. 10.1016/j.neurobiolaging.2007.10.008 18053618

[B50] Suk H.-I. Lee S.-W. Shen D. (2015). Latent feature representation with stacked auto-encoder for AD/MCI diagnosis. Brain Struct. Funct. 220, 841–859. 10.1007/s00429-013-0687-3 24363140 PMC4065852

[B51] Suk H.-I. Shen D. (2013). Deep learning-based feature representation for AD/MCI classification. Med. Image Comput. Comput. Assist. Interv. 16, 583–590. 10.1007/978-3-642-40763-5_72 24579188 PMC4029347

[B52] Suk H.-I. Wee C.-Y. Lee S.-W. Shen D. (2016). State-space model with deep learning for functional dynamics estimation in resting-state fMRI. Neuroimage 129, 292–307. 10.1016/j.neuroimage.2016.01.005 26774612 PMC5437848

[B53] Supekar K. Menon V. Rubin D. Musen M. Greicius M. D. (2008). Network analysis of intrinsic functional brain connectivity in Alzheimer’s disease. PLoS Comput. Biol. 4:e1000100 10.1371/journal.pcbi.1000100 18584043 PMC2435273

[B54] Sylvester C. M. Shulman G. L. Jack A. I. Corbetta M. (2009). Anticipatory and stimulus-evoked blood oxygenation level-dependent modulations related to spatial attention reflect a common additive signal. J. Neurosci. 29, 10671–10682. 10.1523/JNEUROSCI.1141-09.2009 19710319 PMC3417151

[B55] Terry A. V. Jr. Buccafusco J. J. (2003). The cholinergic hypothesis of age and Alzheimer’s disease-related cognitive deficits: recent challenges and their implications for novel drug development. J. Pharmacol. Exp. Ther. 306, 821–827. 10.1124/jpet.102.041616 12805474

[B56] Wang K. Jiang T. Liang M. Wang L. Tian L. Zhang X.. (2006). Discriminative analysis of early Alzheimer’s disease based on two intrinsically anti-correlated networks with resting-state fMRI. Med. Image Comput. Comput. Assist. Interv. 9, 340–347. 10.1007/11866763_42 17354790

[B57] Wang L. Zang Y. He Y. Liang M. Zhang X. Tian L.. (2006). Changes in hippocampal connectivity in the early stages of Alzheimer’s disease: evidence from resting state fMRI. Neuroimage 31, 496–504. 10.1016/j.neuroimage.2005.12.033 16473024

[B58] Zhou J. Greicius M. D. Gennatas E. D. Growdon M. E. Jang J. Y. Rabinovici G. D.. (2010). Divergent network connectivity changes in behavioural variant frontotemporal dementia and Alzheimer’s disease. Brain 133, 1352–1367. 10.1093/brain/awq075 20410145 PMC2912696

